# How does person-centered maternity care relate to postpartum contraceptive counseling and use? Evidence from a longitudinal study of women delivering at health facilities in Ethiopia^☆^

**DOI:** 10.1016/j.conx.2024.100109

**Published:** 2024-08-15

**Authors:** Elizabeth K. Stierman, Celia Karp, Jiage Qian, Solomon Shiferaw, Assefa Seme, Mahari Yihdego, Saifuddin Ahmed, Andreea A. Creanga, Linnea A. Zimmerman

**Affiliations:** a Department of International Health, Johns Hopkins Bloomberg School of Public Health, Baltimore, MD, United States; b Department of Population, Family, and Reproductive Health, Johns Hopkins Bloomberg School of Public Health, Baltimore, MD, United States; c School of Public Health, Addis Ababa University, Addis Ababa, Ethiopia; d PMA-Ethiopia, Addis Ababa University, Addis Ababa, Ethiopia; e Department of Gynecology and Obstetrics, Johns Hopkins Medicine, Baltimore, MD, United States

**Keywords:** Ethiopia, Maternity care, Postpartum family planning, Service integration

## Abstract

**Objectives:**

This study examines the relationship between integrated, person-centered maternity care (PCMC), the provision of postpartum family planning (PPFP) services, and postpartum contraceptive use among women delivering at health facilities in Ethiopia.

**Study design:**

We analyze 2019–2021 longitudinal data from a representative sample of pregnant and recently postpartum women in Ethiopia. This study examines baseline, 6-week, and 6-month survey data collected from women who delivered at a health facility.

**Results:**

Maternity patients who reported more person-centered care were more likely to be counseled on postpartum contraceptive methods before discharge. Overall, 27.5% of women delivering in a health facility received family planning counseling before discharge, ranging from 15.2% in the lowest PCMC quintile to 36.3% in the highest PCMC quintile. The receipt of PPFP counseling was associated with increased odds of postpartum contraceptive use.

**Conclusions:**

Findings suggest dimensions of quality care are interlinked, and person-centered care is associated with greater integration of recommended PPFP services into predischarge procedures. However, even among women who report relatively high levels of person-centered care, our results highlight that family planning is not routinely discussed prior to discharge from delivery, and very few women receive a contraceptive method or referral prior to discharge.

**Implications:**

While most postpartum women report they wish to limit or space future pregnancies, the uptake of modern contraceptive methods in the postpartum period is low. As women increasingly opt to deliver in health facilities, further integration of family planning services into predischarge procedures within maternity care can improve contraceptive access.

**Data statement:**

The data used in these analyses were collected as part of the PMA Ethiopia study. Data are publicly available at https://www.pmadata.org/data/request-access-datasets.

## 1 Introduction

Integration of family planning and maternal health services can increase access to contraception among postpartum women and help prevent unintended and closely spaced pregnancies following childbirth [1]. In Ethiopia, contraceptive use after childbirth is low; 38% of postpartum women in a 2019–2020 national survey reported using contraception at 6-month postpartum [2]. As women increasingly opt to deliver in health facilities, predischarge postpartum family planning (PPFP) counseling offers an important opportunity to improve contraceptive access. However, PPFP counseling is not uniformly provided. Previous research in Ethiopia indicates that first-time mothers and women who have never used contraception are less likely to be counseled on PPFP before discharge from delivery [3].

Successful integration of PPFP services into maternity care likely depends on many factors, including maternity care providers’ communication skills and responsiveness to patients’ preferences, needs, and values. These key attributes of person-centered maternity care (PCMC) are important for a positive childbirth experience and establishing rapport between patients and providers [4]. Similarly, we theorize that counseling on intimate issues, such as PPFP, is more likely to occur—and to be more effective in helping people reach their reproductive goals—in an environment of respect, trust, and strong interpersonal communication. This study investigates the relationship between PCMC, PPFP counseling before discharge from delivery stay, and contraceptive use in the postpartum period among women who delivered at health facilities in Ethiopia.

## 2 Materials and methods

We analyzed data collected between October 2019 and January 2021 from a representative sample of pregnant and postpartum women residing in six regions of Ethiopia, which cover approximately 90% of the country’s population: Addis Ababa, Afar, Amhara, Oromia, Southern Nations, Nationalities, and Peoples’ Region, and Tigray. Sampling followed a multistage procedure [5]. We selected enumeration areas using stratified random sampling and invited pregnant or recently postpartum women aged 15–49 years residing in sampled areas to enroll in the study. In total, 32,628 households (99.1%) in enumerated areas completed the census, and 99.1% of female household members completed the screening questionnaire to determine their eligibility based on pregnancy or recent postpartum status [6]. Those eligible received information about the study, and their informed consent was obtained prior to enrollment. Study participants completed a baseline questionnaire after enrollment and participated in follow-up interviews at approximately 6 weeks and 6 months postpartum. This study examines data from the subgroup of women who delivered at a health facility and completed a postpartum interview. Performance Monitoring for Action (PMA) Ethiopia received ethical approval from Addis Ababa University, College of Health Sciences (Ref: AAUMF 01-008) and the Johns Hopkins University Bloomberg School of Public Health Institutional Review Board (FWA00000287).

We examine the association between PCMC during facility childbirth, receipt of family planning services following childbirth and before discharge from the facility, and contraceptive use in the first 6 weeks and 6 months after childbirth. Measures were ascertained at three time points. The baseline interview collected information about sociodemographics and the participant’s desire to limit or space future pregnancies. The 6-week interview captured information about the participant’s recent experience giving birth at the facility and the care received at the facility immediately after childbirth. This included measures of PCMC, receipt of PPFP counseling prior to discharge, receipt of a contraceptive method or referral prior to discharge, place of delivery, provider attending the delivery, mode of delivery, birth outcomes, and any complications reported during delivery. Participants were asked about current contraceptive use during both the 6-week and 6-month interviews.

We measured PCMC using a validated 13-item patient-reported PCMC scale [7]. This scale, developed by Afulani and colleagues, measures dignity and respect, communication and autonomy, and supportive care during facility childbirth. The 13-item short PCMC scale is a modified version of a longer 30-item scale validated in Kenya, India, and Ghana [8], [9], [10]. Multivariate imputation using chained equations was applied to impute missing PCMC values (<3% per item) [11] based on sociodemographic information and mode of delivery. Scores were converted into quintiles to improve interpretation.

Simple and multiple logistic regression models estimated the relative odds of (1) receiving PPFP counseling prior to discharge for women reporting different levels of PCMC, and (2) contraceptive use at 6-week postpartum and 6-month postpartum according to women’s receipt of PPFP services—specifically, whether she received PPFP counseling prior to discharge (model 1), received a contraceptive method or referral prior to discharge (model 2), or received PPFP counseling at any time postpartum (model 3). Stratified analyses and the addition of an interaction term in models 1 and 2 examined whether the effect of predischarge PPFP services on contraceptive use was modified by PCMC level. Sensitivity analyses reran models, replacing quintiles with a continuous measure of PCMC. We weighted analyses and adjusted variance estimates to account for the complex survey design and variability between imputations.

All multiple regression models adjusted for socioeconomic characteristics (age, education, religion, marital status, parity, wealth quintile, region, urban/rural), characteristics of the delivery (self-reported complications, birth outcome, vaginal vs cesarean delivery, place of delivery, and type of provider attending delivery). Multiple regression models estimating the odds of contraceptive use were adjusted for all the above, plus the woman’s reported desire to limit or space future pregnancies and, at 6 months, whether her menstrual cycle had returned.

## 3 Results

Among eligible pregnant and postpartum women, 2855 (99.6%) enrolled in the PMA-Ethiopia study and completed a baseline questionnaire. This study examines data from the subgroup of women who delivered at a health facility (*n* = 1575, Table 1). Women were followed through 6 months postpartum, with 157 (10% of the facility birth subgroup) lost to follow-up.

**Table 1:** Characteristics of women enrolled in PMA-Ethiopia panel who completed a 6-week postpartum interview and who delivered in a health facility (*n* = 1575)

	Mean (range)^a^
Age, years	26.4 (15–47)
	Count (percentage)^a^
Parity	
0 (Nulliparous)	405 (25.7%)
1–2 Previous births	678 (43.1%)
3–4 Previous births	277 (17.6%)
5+ Previous births	215 (13.6%)
Education	
Never attended	451 (28.6%)
Primary	647 (41.1%)
Secondary	275 (17.5%)
Higher	202 (12.8%)
Wealth quintile	
Lowest	165 (10.5%)
Lower	222 (14.1%)
Middle	288 (18.3%)
Higher	352 (22.3%)
Highest	548 (34.8%)
Religion	
Orthodox	719 (45.7%)
Protestant	368 (23.4%)
Muslim	464 (29.5%)
Other	24 (1.5%)
Currently married^b^	1540 (97.8%)
Region	
Tigray	145 (9.2%)
Afar	9 (0.6%)
Amhara	341 (21.7%)
Oromia	639 (40.6%)
SNNPR^c^	327 (20.8%)
Addis Ababa	114 (7.2%)
Urban residence	608 (38.6%)
Experienced a stillbirth^d^	29 (1.8%)
Had a cesarean delivery	159 (10.1%)
Self-reported complications: delivery or 24 h after	799 (50.7%)
Place of delivery	
Government hospital	594 (37.8%)
Government health center	911 (58.0%)
Government health post	18 (1.2%)
Private facility^e^	51 (3.3%)
Provider attending delivery	
Doctor	304 (19.3%)
Nurse/midwife	699 (44.4%)
Other attendant/cannot distinguish	573 (36.4%)
Desire to limit or space future pregnancies	
Does not want more children^f^	273 (17.3%)
Wants next child in 2+ y or undecided^g^	1276 (81.0%)
Wants next child in <2 y	27 (1.7%)

CI, confidence interval; NA, not applicable; SNNPR, Southern Nations, Nationalities, and Peoples' Region.

a Reflects weighted mean, weighted count, and weighted percentage; estimates weighted to account for complex survey design; categories may not sum to 1575 due to rounding.

b Currently married or living together as if married.

c SNNPR was subsequently divided into three regions: SNNPR, Sidama, and South West Ethiopia People’s Region; results are representative of political boundaries in September 2019.

d In case of multiple births, categorized as stillbirth if any of the births were stillbirths. This reflects the percentage of women in the sample who experienced a stillbirth (not the stillbirth rate).

e Includes facilities managed by private for-profits, nongovernmental organizations, and faith-based organizations.

f At baseline, wanted no more children (if recently postpartum) or wanted no more children after current pregnancy (if currently pregnant). For one respondent with missing value at baseline, response reflects answer to same question asked at 6-month interview.

g At baseline, wanted to wait 2 or more years for next child, wanted another child but undecided on timing, or undecided if wanted another child. For five respondents who wanted another child but were missing a value for preferred timing at baseline, responses reflect preferred timing at 6-month interview.

**Fig. 1:**
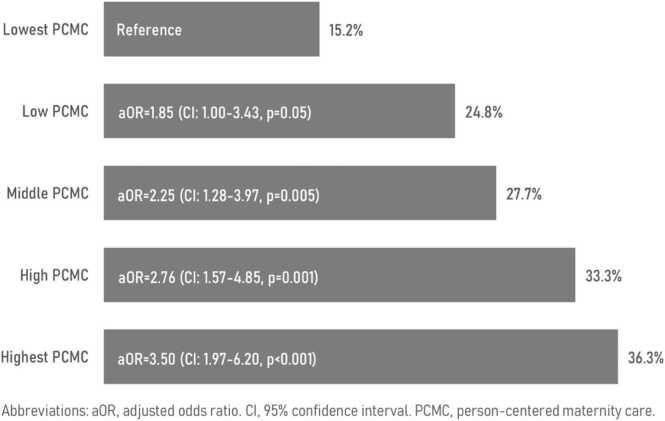
Receipt of predischarge family planning counseling after facility childbirth by level of PCMC, Ethiopia, 2019–2021.

Overall, 27.5% of women delivering in a health facility received family planning counseling before discharge, ranging from 15.2% in the lowest PCMC quintile to 36.3% in the highest PCMC quintile (Fig. 1). Compared to those in the lowest PCMC quintile, respondents in the middle, higher, and highest PCMC quintile (i.e., whose maternity care experiences were more person-centered) had 2.25 (95% confidence interval [CI]: 1.28–2.97), 2.76 (95% CI: 1.57–4.85), and 3.50 (95% CI: 1.97–6.20) times higher adjusted odds of receiving family planning counseling prior to discharge. In sensitivity analyses, an increase in PCMC, measured on a continuous scale, remained significantly associated with higher adjusted odds of family planning counseling prior to discharge.

**Fig. 2:**
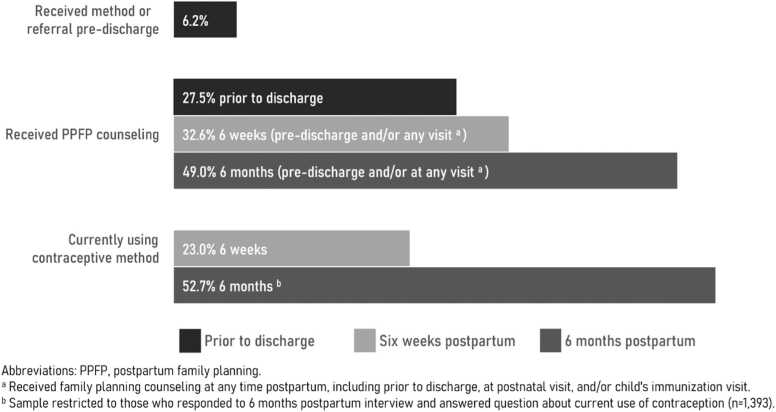
Receipt of PPFP services and contraceptive use among postpartum women who delivered at a health facility in Ethiopia, 2019–2021.

Among our sample of postpartum women who delivered at a health facility, the percentage using a contraceptive method was 23.0% at 6-week and 52.7% at 6-month postpartum (Fig. 2). The most common contraceptive methods used in our sample were injectables (53.7%), implants (25.5%), and pills (10.4%; Table 2). At 6-week postpartum, the adjusted odds of contraceptive use were 1.65 (95% CI: 1.15–2.36) times greater for those who received family planning counseling before discharge; 1.68 (1.23–2.29) times greater for those who received family planning counseling at some point during the postpartum period; and 10.4 (5.0–21.6) times greater for those who received a method or referral before discharge than those who did not (Table 3). At 6-month postpartum, receipt of family planning counseling at any time during the postpartum period (adjusted odds ratio: 2.12; 95% CI: 1.41–3.17) and receipt of a method/referral before discharge (adjusted odds ratio: 5.39; 95% CI: 2.15–13.50) remained significant predictors of contraceptive use. There was no evidence that the level of PCMC modified the effect of PPFP counseling on contraceptive use.

**Table 2:** Contraceptive methods used at 6 months postpartum, among postpartum women who delivered at a health facility in Ethiopia, 2019–2021

	Currently using a method at 6 mo (*n* = 801)		Timing can generally start using method per WHO recommendations for postpartum family planning^a^	
	Count	Percentage (95% CI)	Breastfeeding	Nonbreastfeeding
Injectables	430	53.7 (48.2–59.2)	≥6 wk (progestogen-only, DMPA, and NET-EN^b^)	Immediately postpartum (progestogen-only, DMPA, and NET-EN)
Implant	204	25.5 (21.2–29.7)	Immediately postpartum (progestogen-only, LNG, and ETG)	
Pill	84	10.4 (7.7–13.2)	Immediately postpartum (progestogen-only, POP)	
LAM	34	4.2 (1.9–6.5)	Immediately postpartum	Not applicable
Other^c^	49	6.2 (3.8–8.6)	-	-

CI, confidence interval; DMPA, deport medroxyprogesterone acetate; ETG, etonogestrel; LAM, lactational amenorrhea method; LNG, levonorgestrel; NET-EN, norethisterone enanthate; POP, progestogen-only pill.

If respondent reported use of more than one method, most effective method is shown.

a WHO postpartum family planning compendium. World Health Organization. Available at https://postpartumfp.srhr.org/ (accessed June 28, 2023) and Medical Eligibility for Contraceptive Use, Fifth Edition. World Health Organization; 2015.

b Recommendations shown for progestogen-only contraceptives, including progestogen-only pills, progestogen-only injectables, and levonorgestrel and etonogestrel implants. For combined hormonal contraceptives including combined injectable contraceptives, combined oral contraceptives, combined contraceptive patches, and combined vaginal rings, WHO recommends that breastfeeding women generally start using combined hormonal contraceptives at ≥6 months postpartum and nonbreastfeeding women at ≥3 weeks postpartum.

c Other methods include rhythm method (2.9%), withdrawal (1%), emergency contraception (<1%), standard days/cycle beads (<1%), male condom (<1%), intrauterine device (<1%), and female sterilization (<1%).

**Table 3:** Odds of contraceptive use given receipt of postpartum family planning services, among postpartum women who delivered at a health facility in Ethiopia, 2019–2021

	Contraceptive use at 6 wk postpartum (*n* = 1575)		Contraceptive use at 6 mo postpartum (*n* = 1393^a^)	
	OR (95% CI)	aOR^b^ (95% CI)	OR (95% CI)	aOR^c^ (95% CI)
Model 1: Received family planning counseling before discharge from birthing facility (ref=none)	1.73 (1.22–2.45)^d^	1.65 (1.15–2.36)^d^	1.33 (0.97–1.82)	1.38 (0.95–2.00)
Model 2: Received family planning method or referral before discharge from birthing facility (ref=none)	7.86 (3.85–16.0)^e^	10.4 (5.0–21.6)^e^	3.31 (1.54–7.09)^d^	5.39 (2.15–13.50)^e^
Model 3: Received family planning counseling at any time postpartum^f^ (ref=none)	1.78 (1.31–2.43)^e^	1.68 (1.23–2.29)^d^	1.83 (1.32–2.52)^e^	2.12 (1.41–3.17)^e^

aOR, adjusted odds ratio; CI, confidence interval; OR, odds ratio.

a Sample excludes those lost to follow-up at 6 months (*n* = 157) and those who did not respond to the question on contraceptive use at 6 months (*n* = 25).

b Adjusted for socioeconomic characteristics (age, education, religion, marital status, parity, wealth quintile, region, urban/rural), characteristics of the delivery (self-reported complications, birth outcome, vaginal vs cesarean delivery, place of delivery, and type of provider attending delivery), and desire to limit or space future pregnancies.

c Adjusted for all above and whether menstrual cycle had returned by 6 months interview.

d *p* < 0.01.

e *p* < 0.001.

f Received family planning counseling at any time postpartum, including prior to discharge, at postnatal visit, and/or child’s immunization visit.

## 4 Discussion

While nearly all respondents wanted to limit or space future pregnancies (Table 1), only half reported using a contraceptive method at 6-month postpartum. Integrating PPFP services into predischarge procedures within maternity care offers an opportunity to reach women early in the postpartum period, improve understanding of women’s return to fertility, and provide postpartum women with contraceptive options on-demand.

We find that women who reported more person-centered care were more likely to be counseled on postpartum contraceptive methods predischarge. While our data do not allow us to comment on the directionality of this relationship, we observed an incremental relationship, wherein higher PCMC scores were associated with greater odds of counseling. Consistent with the World Health Organization’s Quality of Care Framework [12], findings suggest dimensions of quality care are interlinked, and person-centered care connects with providers’ adherence to recommended practices, such as postpartum counseling on family planning.

At the same time, our results highlight that PPFP is not routinely discussed prior to discharge from delivery, even among women who report relatively high levels of person-centered care. Even fewer women receive a PPFP method or referral prior to discharge. This is a missed opportunity given the high proportion of women who wish to limit or space future pregnancies. Based on World Health Organization recommendations, women may safely use a wide variety of contraceptive methods, including progestogen-only pills and implants, immediately after birth [13], [14]. Nonbreastfeeding women can also use progestogen-only injectables immediately after birth, while breastfeeding women can safely begin use at 6 weeks postpartum [13], [14]. Implants, pills, and injectables are the most common contraceptive methods used in Ethiopia [15] and by our sample of postpartum women (Table 2). However, women do not always receive counseling that these methods can be used safely immediately postpartum.

Consistent with other studies [16], we find that women who receive PPFP services as part of childbirth care are significantly more likely to use modern contraceptive methods in the postpartum period. This is especially true for those women who received a contraceptive method or referral for a method prior to discharge from the birthing facility. Increasingly, in diverse contexts, facilities are proactively offering contraceptive methods and referrals prior to discharge [16]. This can facilitate timely and safe access to contraception when a return visit may be costly or inconvenient. National guidelines for family planning services in Ethiopia recommend providers introduce immediate postpartum contraceptive options to women after delivery, but our findings suggest this practice is not routinely implemented [17].

This study sought to understand the influence PCMC may have on PPFP services and use. We theorized that a respectful, supportive environment may influence the quality of counseling and the uptake of contraceptive methods to support a woman’s reproductive goals. Although we did find that women who reported more person-centered care were more likely to receive PPFP counseling, we did not find evidence that PCMC modified the effectiveness of counseling on contraceptive use. This finding is difficult to interpret without more nuanced information on the knowledge, skills, and attitudes of maternity care providers concerning PPFP, the availability of PPFP methods at birthing facilities, or the extent that PPFP is integrated into protocols and standard practices adopted in facilities. In the absence of supportive conditions, even the most attentive and communicative maternity care providers may be limited in their ability to offer quality PPFP services. More research, particularly in-depth qualitative research, could help provide a more nuanced understanding of the barriers and facilitators to integration of immediate PPFP into childbirth care—and how these services can better help women meet their family planning goals.

Limitations of the study include its reliance on self-reported information, which could be prone to recall and other response bias. Data on participants’ experience giving birth and the care received at the birthing facility were obtained during a 6-week postpartum interview conducted at the participant’s home. By conducting interviews at the respondents’ home, this design aimed to minimize bias that may arise when interviews are conducted in facilities, and patients worry that negative responses could affect their care. However, participants may have greater difficulty remembering previous events with accuracy. Moreover, differences may exist between groups in their likelihood to recall specific events. For example, those who are currently using a contraceptive method may be more likely to recall receiving PPFP services than those who are not, and those who received a contraceptive method prior to discharge may be more likely to recall receipt of PPFP counseling than those who did not. This could account, or partially account, for the observed association between these variables.

Our study analyzes data from women who gave birth at a health facility and exclude those who gave birth at home. Thus, our sample includes a greater proportion of urban and wealthier women relative to the general population in Ethiopia. Postpartum contraceptive use tends to be higher among these groups [2]. Nevertheless, our results suggest that unmet needs for contraception exist in our study population despite greater use of maternity and reproductive health care services. These findings have important implications in the Ethiopian context, where increasingly more women deliver in facilities, underlining the need for further integration of maternity and family planning services.

## Author contributions

A.A.C. and S.A.: writing – review & editing, methodology, conceptualization; L.A.Z., S.S., and A.S.: writing – review & editing, supervision, project administration, methodology, funding acquisition, conceptualization; M.Y.: writing – review & editing, supervision, project administration, data curation; C.K. and J.Q.: writing – review & editing, writing – original draft, methodology, formal analysis, conceptualization; E.K.S.: writing – review & editing, writing – original draft, visualization, methodology, formal analysis, conceptualization.

## Declaration of Competing Interest

The authors declare that they have no known competing financial interests or personal relationships that could have appeared to influence the work reported in this article.
